# Axillary Lymph Node Metastasis in Medullary Thyroid Carcinoma: A Case Report

**Published:** 2017-09

**Authors:** Seyed Ziaeddin Rasihashemi, Ramin Azhough, Ali Ramouz

**Affiliations:** 1 *Department of Cardiothoracic Surgery, Imam Reza Hospital, Tabriz University of Medical Sciences, Tabriz, Iran.*; 2 *Department of General Surgery, Imam Reza Hospital, Tabriz University of Medical Sciences, Tabriz, Iran.*

**Keywords:** Lymph node, Metastasis, Neck dissection, Thyroid neoplasms.

## Abstract

**Introduction:**

Medullary thyroid cancer (MTC) is an uncommon neoplasm originating from parafollicular C cells. Distant metastasis in MTC, such as axillary node involvement, is extremely rare.

**Case Report:**

The present study describes a known case of MTC with axillary lymph node metastasis in a 31-year-old woman. In 2010, she underwent total thyroidectomy and right-sided modified radical neck dissection. In May 2015, she was referred with a 3-month history of a mass in the left axilla. Fine needle aspiration cytology (FNAC) confirmed MTC in the axillary nodes. Left axillary lymph node dissection was performed and postoperative histopathology revealed metastatic medullary thyroid carcinoma in prepared specimens.

**Conclusion:**

MTC with axillary lymph node metastasis is a rare condition which has been reported in previous studies to impair patient prognosis. However, in the current case, the patient had no other MTC-related complications subsequent to final lymphadenectomy.

## Introduction

Medullary thyroid cancer (MTC) is an uncommon neoplasm originating from thyroid parafollicular C cells and accounting for approximately 3% of thyroid gland cancers (1–3). Fine needle aspiration cytology (FNAC), serum calcitonin level, RET protooncogene testing, and carcinoembryonic antigen (CEA) level are used to diagnose MTC preoperatively (1,3). However, approximately 10–15% of MTC diagnoses are made after thyroidectomy during pathological studies (1).

A number of studies have shown that early diagnosis is affirmative and improves the 10-year survival rate to approximately 95% (3). However, 10-year survival rate among MTC patients with distal metastasis has been reported to be only approximately 40–50% (4,5).

Distant metastasis such as axillary node involvement is a rare condition that has been reported in only 20% of MTC patients (2,4).In the context of this background, in the current report we describe an MTC patient with uncommon metastasis in the axillary lymph nodes.

## Case Report

In May 2015, a 31-year-old women with a history of medullary thyroid cancer presented with a 3-month history of a mass in the left axilla. She had undergone a total thyroidectomy with right-sided cervical lymphadenectomy 5 years previously, and had been investigated for multiple endocrine neoplasia. No mutation was associated with the familial forms of MTC according to genotype screening. 

Two years later, the patient developed a recurrence of cervical lymphadenopathy, for which she underwent left-sided modified radical neck dissection and superior mediastinal lymphadenectomy because of several hypoechoic lymph nodes detected during ultrasonography examination.

Physical examination revealed a solid, mobile, painless mass, which was approximately 5 × 7 cm in size in the left axilla. 

All laboratory tests results, including T_4_, thyroid-stimulating hormone (TSH) and T_3_, were normal with the exception of serum calcitonin level which was reported to be 1,383 pg/ml (N: <10 pg/ml). Ultrasonography showed at least five lymph nodes with irregular margins and hypoechoic centers in the left axillary. A whole body scan with mTc_99_-octreotide showed abnormal increases in uptakes at the left axillary, with a remarkable size enlargement. FNAC was carried out on the axillary nodes and confirmed MTC.

**Fig 1:**
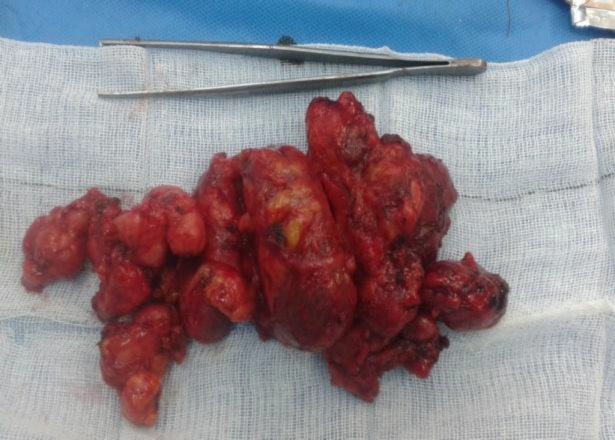
Macroscopic illustration of axillary lymph nodes

**Fig 2:**
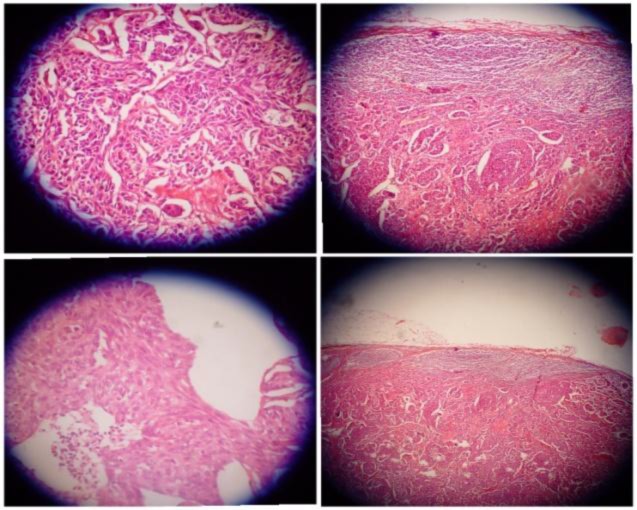
Axillary lymph nodes with MTC metastasis microscopic specimens

The patient underwent dissection of the left axillary lymph nodes. Lymphadenopathy was detected at levels I and II without further extension (Fig. 1). There was no involvement of the vessels or large nerves, including long thoracic or thoracodorsal nerves. Postoperative histopathology confirmed metastatic medullary thyroid carcinoma in the resected lymph nodes (Fig. 2).

The patient was discharged from the hospital on the fifth postoperative day. During the current period of follow-up, the patient has shown no signs or symptoms. For further treatment, the patient was referred to an oncologist.

## Discussion

The term thyroid cancer encompasses various types of tumor with significantly different characteristics, due to differences in the origin of the tumor cells. MTC is a rare condition originating from thyroid gland C cells that secrete calcitonin, and accounts for approximately 5–10% of all thyroid cancers (3,5). Calcitonin is a specific and sensitive biomarker in MTC diagnosis. On the other hand, higher CEA levels are associated with a higher rate of undifferentiating among C cells and poorer prognosis. Therefore, these markers can be helpful not only in the diagnostic procedure but also in the follow-up of patients.

Although initial MTC staging has reported an incidence of 80% of pathologically proven cervical lymph node metastasis (LNM), the role of primary and elective neck dissection is not confirmed (3). The LNM rate is approximately 36% for mediastinal lymph nodes, while axillary LNM is a rare condition occurring in less than 20% MTC patients (2). According to the Rouviere description, there is a physiologic centripetal flow from the axillary mediastinal and cervical lymph nodes to the jugulo-subclavian junction. Ozdemir et al. suggested that blockage in the jugulo-subclavian junction due to metastasis of the cancer or fibrosis as a result of surgical manipulation or radiotherapy can alter the centripetal flow in the junction that leads to a retrograde pathway of lymphatic flow. This may be considered as the underlying reason for distant metastasis, especially axillary LNM (3).

Currently, there are very few reports in the literature that prove the incidence of axillary LNM in MTC patients. Ozdemir et al. demonstrated that axillary LNM is concurrent with distant organ metastasis, and associated with poor prognosis and higher mortality (3). However, we report here that after axillary lymph node dissection, there was no evidence of other metastasis or distant metastasis during the follow-up period. Therefore, we suggest that axillary LNM cannot be considered an indicator for distant metastasis, but that concurrence with distant metastasis may be due to late diagnosis.

Surgical resection is the preferred treatment approach for MTC with axillary LNM. A number of studies have reported the efficacy of kinase inhibitors in the treatment of locally advanced or metastatic MTC (3). We decided to consult with an oncologist regarding the necessity of these drugs.

## Conclusion

Axillary node metastasis from MTC is a very rare condition and may be considered concurrent or recurrent disease. Follow-up is recommended in patients undergoing neck dissection from MTC. However, despite previous reports in the literature, axillary LNM did not diminish the patient’s overall prognosis in the current case, and there were no other report of recurrence or other metastasis during the follow-up period. Therefore, it can be concluded that further studies are needed to investigate the role of axillary LNM in terms of prognosis in MTC.
